# Unintentional Implantation of a Permanent Pacemaker Lead Across a Patent Foramen Ovale Leading to Left Ventricular Pacing

**DOI:** 10.7759/cureus.49277

**Published:** 2023-11-23

**Authors:** Ibrar A Khan, Sajjad Mazhar

**Affiliations:** 1 Department of Cardiology, Southend University Hospital, Mid and South Essex NHS Foundation Trust, Southend-on-Sea, GBR

**Keywords:** lv (left ventricle), anticoagulation, thromboembolism, permanent pacemaker (ppm), patent foramen ovale (pfo)

## Abstract

Unintentional placement of a left ventricular lead through a patent foramen ovale (PFO) is an uncommon and underdiagnosed complication. Normal single- or dual-chamber permanent pacemaker implantation involves placing a lead across the tricuspid valve into the right ventricle. In a very rare case instead of the lead going into the right ventricle, it goes through the PFO and across the mitral valve into the left ventricle (LV) resulting in LV pacing. We describe a case of one of our patients who presented with syncope due to bifascicular block and underwent a dual-chamber pacemaker implantation at a local hospital. He had a background of paroxysmal atrial fibrillation and sarcoidosis. Post-procedure, he was discharged with an inadvertent lead in the LV that was not identified. Abnormal placement of LV leads can result in serious complications including thromboembolism, mitral regurgitation, and left-sided endocarditis. Treatment options include extraction of the lead or anticoagulation.

## Introduction

Dual-chamber pacemaker implantation is a common procedure carried out to prevent syncope in heart block. However, as in all procedures, there are some risks associated with it. As a part of normal post-permanent pacemaker (PPM) check, it is required to do chest X-ray and ECG. Sometimes those investigations are misinterpreted, and complications go unnoticed. Implantation of the left ventricle (LV) through PFO is a rare complication of PPM implantation. A patent foramen ovale (PFO) is a small hole in the interatrial septum that is present in 24-27.5% of the population [1,2]. Early recognition and management are necessary if this complication is identified as it poses a serious outcome.

## Case presentation

**Figure 1:**
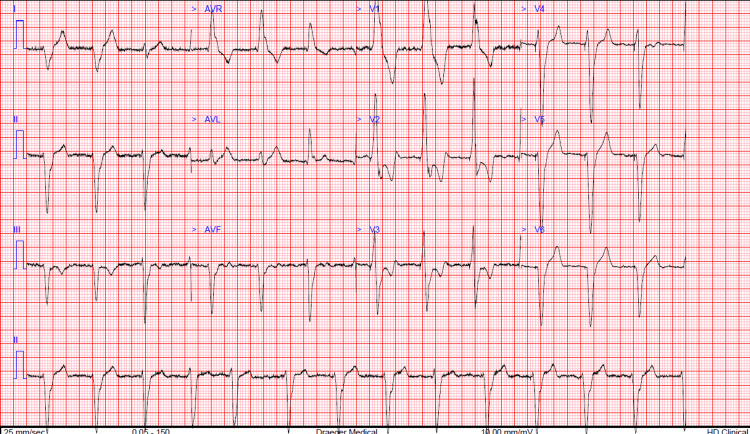
ECG showing paced rhythm with RBBB with pacing spikes with prominent QRS in right-sided precordial leads ECG: Electrocardiogram; RBBB: right bundle branch block pattern

**Figure 2:**
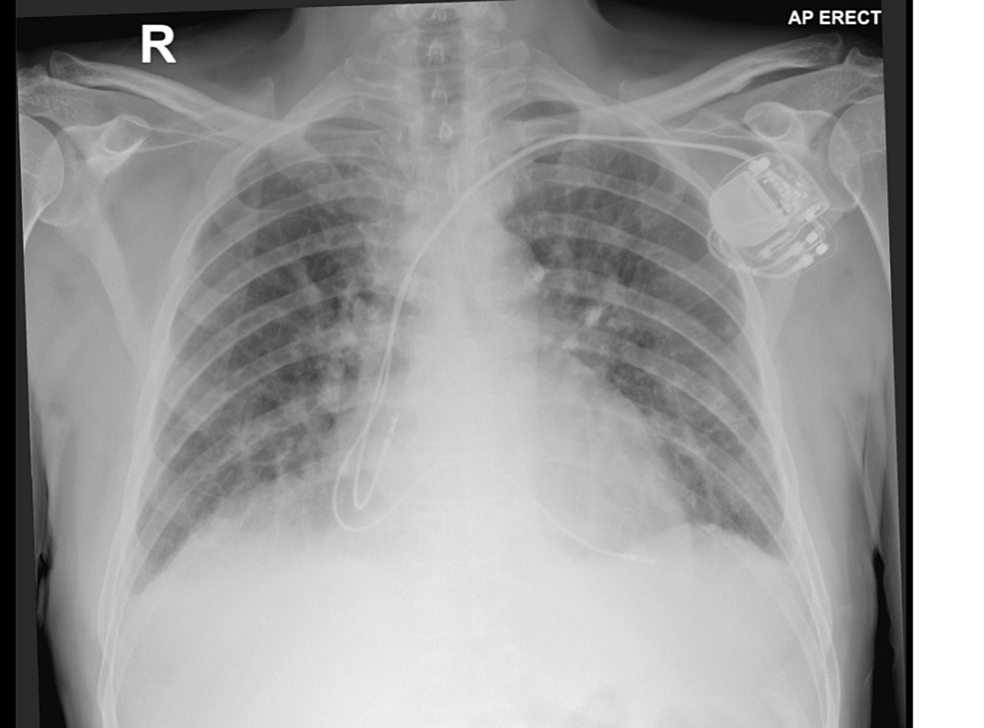
CXR AP view showing the lead in the RA and lead in the LV which is hard to differentiate from the normally positioned RV lead CXR: Chest X-ray; AP: antero-posterior; RA: right atrium; LV: left ventricle; RV: right ventricle

**Figure 3:**
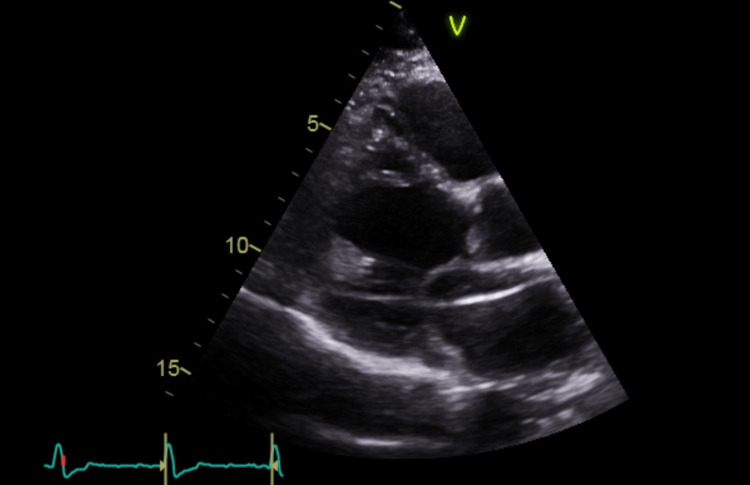
PLAX TTE view showing the lead in the LA going across the mitral valve into the left ventricle PLAX: Parasternal long axis; TTE: transthoracic echocardiogram; LA: left atrium

**Figure 4:**
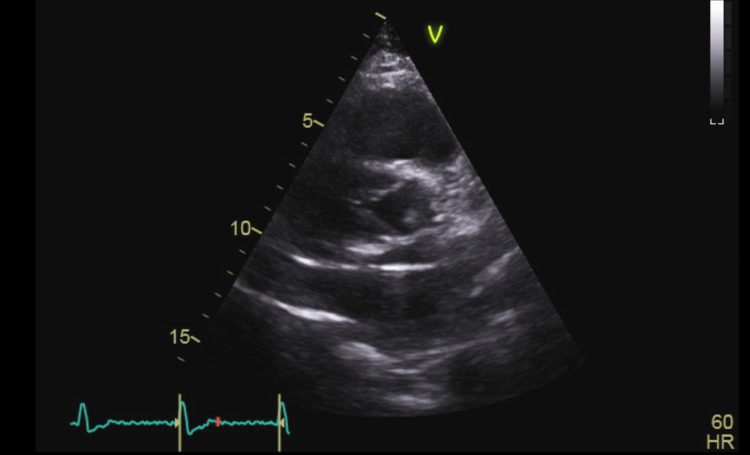
PSAX TTE view at the level of aortic valve with the lead passing through the PFO from the RA to the LA PSAX: Parasternal short axis; TTE: transthoracic echocardiogram; PFO: patent foramen ovale; RA: right atrium; LA: left atrium

**Figure 5:**
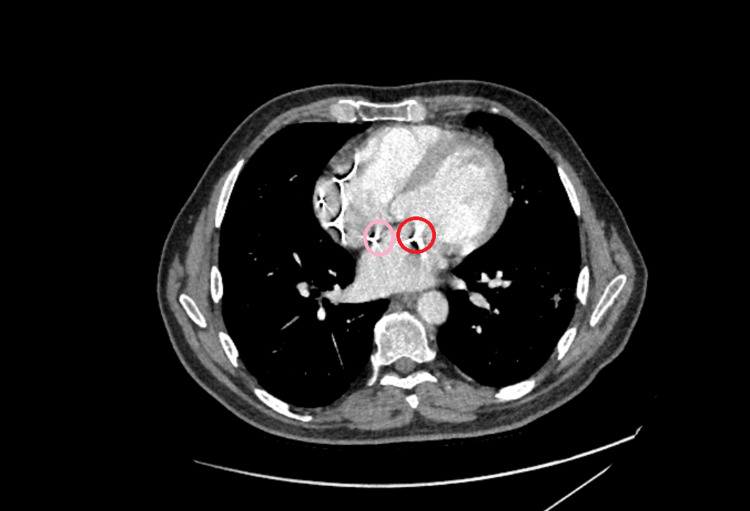
CT chest showing leads blooming artefact due to pacing lead at the level of PFO as indicated by a pink circle and mitral valve as indicated by a red circle CT: Computerised tomography; PFO: patent foramen ovale

A 68-year-old man who had a PPM implantation nine years ago in one of the local hospitals due to underlying bifascicular block and syncope presented to our hospital. His care was shifted to our hospital as the patient moved to a new area and pacing check demonstrated atrial fibrillation (AF) with a right bundle branch block (RBBB) pattern as shown in Figure 1. He was subsequently admitted to the hospital with congestive heart failure and his LV function was severely compromised due to AF with fast ventricular rate. However, his LV function recovered afterwards with good heart rate control and AF cryoablation. The likely reason for ECG changes after pacemaker implantation could be baseline RBBB, placement of RV active fixation on the septum or across the septum into the LV and less commonly placement of lead into coronary sinus or through the PFO into the LV. Initial echo raised a suspicion of an abnormal echogenic structure in LA that is going towards the mitral valve. Further imaging including chest X-ray, transthoracic echocardiogram and cardiac CT demonstrated that lead went through PFO and across the mitral valve into the LV as shown in Figures 2-5. There was no LV thrombus seen on imaging. This complication was recognised seven years after his pacemaker implantation. As the patient was already anticoagulated due to paroxysmal AF six years ago, consensus was made to continue with anticoagulation in the form of rivaroxaban as extraction would have been a complicated procedure with an increased risk of adverse events.

## Discussion

Abnormal lead position across PFO into the LV is a rare complication. It was reported that the incidence of an inadvertent lead position in the left side of the heart, including cardiac veins, is 3.4% [3]. However, early recognition is helpful to prevent complications related to the thromboembolic phenomenon. Identifying abnormal lead positions is possible during the procedure. It is advisable during the procedure to do a left anterior oblique view (LAO) to differentiate the anterior position of the right ventricle and tricuspid valve with posteriorly located LV and mitral valve. Post-procedure chest X-ray lateral view is also important to identify the lead position. Only a postero-anterior view or antero-posterior view of the chest X-ray is not enough to identify this complication. ECG also plays a vital role in identifying abnormal positioning of right ventricle lead [4].

The most serious complication of unintended LV lead implantation is thromboembolism [3] and 42 % of the patients who suffer from stroke/TIA had a device implanted in <12 months [5]. The use of antiplatelets is unlikely to help in this regard. It is reported that a clot can form within two weeks of lead placement with the earliest thromboembolic event reported after one month of implantation [6]. There are two options to deal with this complication. The first one is extraction either percutaneous or surgical. The second option is long-term anticoagulation.

Transcatheter extraction is a feasible option if performed within two weeks of implantation [7]. For those patients who are on antiplatelets, their extraction can be slightly delayed. The transcatheter procedure is associated with increased risk of dislodgement of thrombus resulting in stroke [8]. Surgical extraction carries less risk of thromboembolism with increased morbidity. However, surgical extraction should be considered if there is any other surgical indication such as coronary artery bypass grafting [9]. It is worth mentioning here that it is difficult sometimes to recognise a clot or thrombus attached to the leads even with transoesophageal echocardiography [10].

Regarding anticoagulation, there are some reported cases in which no thromboembolic event occurred for up to 10 years when patients were chronically anticoagulated [11]. There is limited data on the use of direct oral anticoagulants (DOACs) in patients with left ventricular lead implantation. However, recent data shows that DOACs are non-inferior or at least as effective as warfarin in the management of LV thrombus [12].

In short, after one year of lead implantation, the safest option could be long-term anticoagulation [7]. If the post-implantation duration is between a fortnight and one year, then a management plan should be made on a case-by-case basis. As in all complicated cases, the decision should be according to patient wishes, comorbid factors and multi-disciplinary discussion.

## Conclusions

PPM implantation is a common procedure nowadays. As in all procedures, there is always some risk of complications. The presence of PFO is not uncommon and a pacemaker lead can go easily through the PFO into the LV and may increase the risk of thromboembolism. Early periprocedural identification with fluoroscopy or post-procedure investigations such as electrocardiogram and chest X-ray lateral and PA views are important to recognise early pacemaker complications. Especially, if the RBBB pattern is identified post-implantation, the clinician should be alerted. Transthoracic ECG is also helpful if there is any suspicion. Once the complication is identified, the safest option is to reposition the lead as soon as possible particularly within one year of implantation or anticoagulation could be considered if the complication is identified after 12 months. However, the final decision should be made by the multidisciplinary team depending on the case and its co-morbidities.
